# Supplementary figures and images for: Embryo classification beyond pregnancy: early prediction of first trimester miscarriage using machine learning
Source: J Assist Reprod Genet. 2022 Oct 4;40(2):309–22. doi: 10.1007/s10815-022-02619-5 (PMC9935804; doi:10.1007/s10815-022-02619-5)

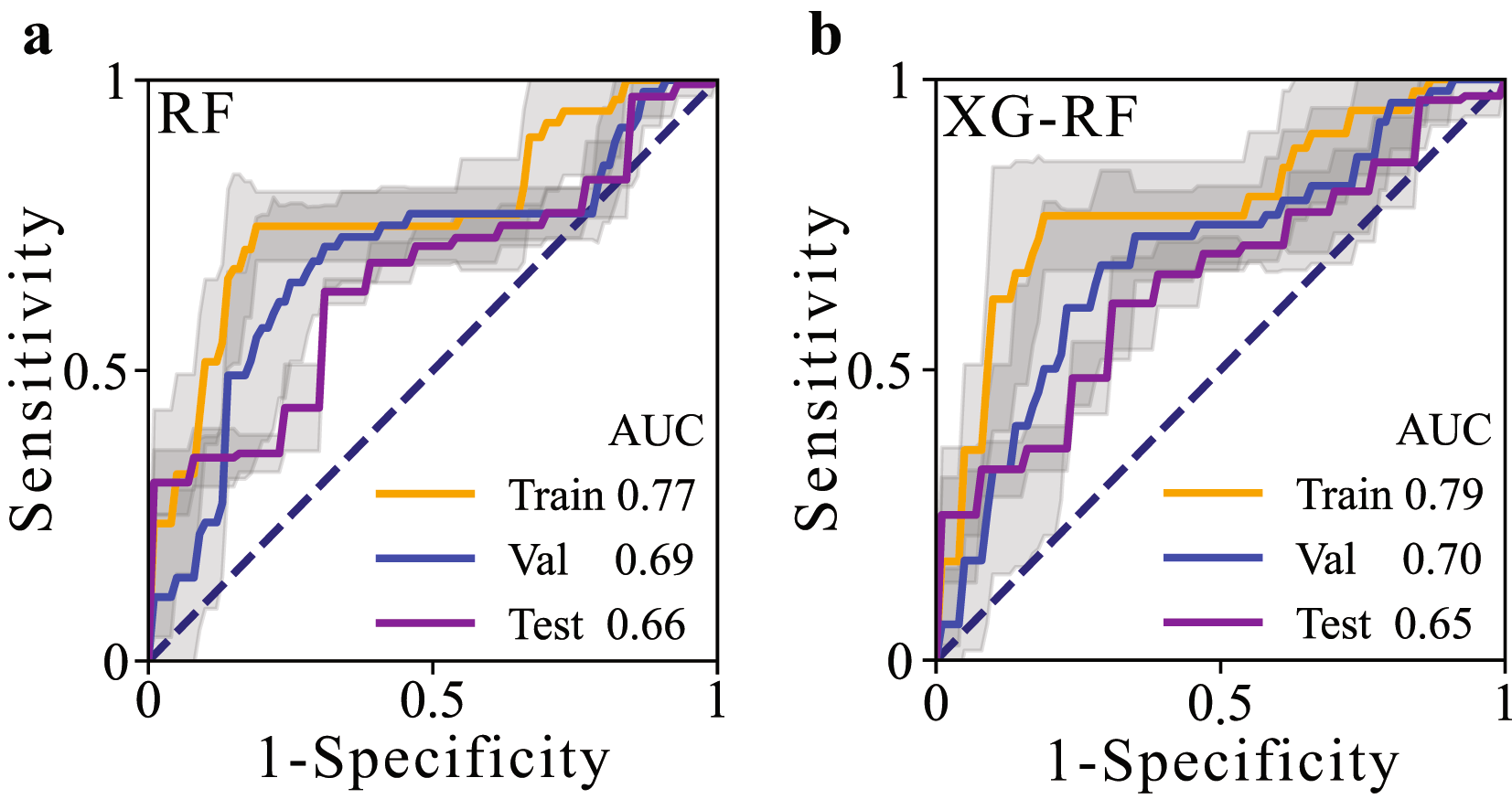

Supplement: Supplementary file 2 — Figure S1: MC prediction by an RF model and an RF-XG integrated model using embryos form H2-to-H4 clinics. Train, validation and test set ROC curves of MC prediction using an RF model (a) and an integrated XG-RF classifier (b). The latter corresponds to averaging the MC scores that were obtained by RF and XG models. RF: Random forest. Here, embryos from H1 clinic were removed. XG: XGBoost. ROC: Receiver operating characteristic. AUC: Area under the ROC curve. (PNG 156 kb) [file 10815_2022_2619_Fig10_ESM.png]
